# Revisiting the impact of race/ethnicity in endometriosis

**DOI:** 10.1530/RAF-21-0106

**Published:** 2022-03-17

**Authors:** Olga Bougie, Ikunna Nwosu, Chelsie Warshafsky

**Affiliations:** 1 Department of Obstetrics and Gynaecology, Queen’s University, Kingston, Ontario, Canada; 2 School of Medicine, Queen’s University, Kingston, Ontario, Canada; 3 Department of Obstetrics and Gynecology, University of Ottawa, Ottawa, Ontario, Canada

**Keywords:** endometriosis, pelvic pain, race, ethnicity

## Abstract

Endometriosis is a chronic, multisystemic disease often presenting with significant phenotypic variation amongst patients. The impact of race/ethnicity on the prevalence of endometriosis, as well as disease presentation, is a question of interest which has been explored for the last century. This narrative review explores the historical perspective of endometriosis and race/ethnicity as well as the evidence available to date. Furthermore, we discuss the potential implication of the bias perpetuated on this topic, specifically in the areas of medical education, research, and clinical care. In consideration of these intersecting realms, we suggest priorities for future consideration of race/ethnicity as it pertains to the delivery of care for endometriosis patients.

**Lay summary:**

The relationship between race/ethnicity and endometriosis has been explored for over a century. Historical bias and poorly conducted research have led to the idea that this condition is less likely to be diagnosed in certain racial groups, such as Black women. We review the current state of evidence and highlight important limitations within medical education and research on this topic. Finally, we advocate for a shifting viewpoint as we strive to deliver equitable and outstanding care for all endometriosis patients.

## Introduction

Endometriosis is a chronic, multisystemic disease of inflammation affecting approximately 10% of the female population (Eskenazi & Warner 1997, Zondervan *et al.* 2018, 2020). It is defined as the extra-uterine growth of endometrial glands and stroma and may present with a variety of symptoms such as pelvic pain and infertility, resulting in a significant negative impact on individuals’ health and quality of life (Nnoaham *et al.* 2011, Zondervan *et al.* 2018, Missmer *et al.* 2021). The most widely regarded theory on the origin of endometriosis dates back to Dr Sampson, who postulated that endometriotic implants resulted from retrograde menstruation back in the 1920s, although reports of aberrant endometrial tissue growth date back to as early as 1860 (Sampson 1921, Counseller 1938, Sanmiguel 2000: 80–134, Benagiano *et al.* 2014).

## Historical perspective

John A Sampson was interested in exploring the reason for infertility seen in patients with endometriosis, at a time of social concern regarding declining birth rates among upper-class women in the United States (Marsh & Ronner 1996, Sanmiguel 2000: 80–134, Gordon 2002). In the context of such societal panic, Dr J Meigs proposed a theory that endometriosis was linked to contraceptive use and delayed childbearing seen most commonly in the ‘well-to-do’ (Meigs 1938). This theory gained ground for several decades, substantiated by methodologically flawed research demonstrating increased rates of endometriosis among private White patients compared to the ward Black patient, a dichotomy ridden with confounding and bias (Meigs 1941, Scott & TeLINDE 1950, Blinick & Merendino 1951, Weed 1955). Although some evidence to the contrary started to emerge in the 1950s, it was not until Dr Chatman presented his work in the 1970s that the view of low prevalence of endometriosis in Black patients began to shift (Chatman 1976). Nevertheless, by this point, a strong bias regarding the impact of race/ethnicity in the epidemiology of endometriosis was perpetuated in the medical community, evidenced by the narrative in medical education literature suggesting a rarity of endometriosis amongst non-white patients, present until the twentieth century (Hayden 1956, Novak *et al.* 1961: 247–250, Kistner 1971: 432–456, Speroff *et al.* 1983: 495–503, Fritz & Speroff 2005: 1103–1114). More recent texts continue to suggest lower prevalence of endometriosis diagnosis in Black and potentially higher prevalence among Asians, compared to White women (Fritz & Speroff 2011: 1221–1233, Hoffman 2020).

## Summary of evidence regarding race/ethnicity and endometriosis

Several publications dating back to the 1920s have investigated the epidemiological risk factors for developing endometriosis, including race/ethnicity. This literature was synthesized in a systematic review and meta-analysis by Bougie *et al.* (2019*b*) in order to estimate the risk of endometriosis among various racial/ethnic groups. The review included 18 studies and identified that compared to White women, Black and Hispanic woman were less likely to be diagnosed with endometriosis (Black women - OR: 0.49, 95% CI: 0.29–0.83; Hispanic women -, OR: 0.46, 95% CI: 0.14–1.50), while Asian women were more likely to have this diagnosis (OR: 1.63, 95% CI: 1.03–2.58). Significant heterogeneity was present in the analysis for all racial/ethnic groups, which may have stemmed from clinical variation of included study participants and definition of outcomes, as well as methodological differences in study design. Two studies were not included in the meta-analysis as they reported outcomes of interest in a format not compatible with data synthesis. First, Missmer *et al.* (2004) examined the incidence of surgically diagnosed endometriosis in the Nurse’s Health Study II and found that Black women had lower rate of endometriosis diagnosis compared to White women (RR: 0.6, 95% CI: 0.4–0.9), whereas Asian (RR: 0.8, 95% CI: 0.5–1.1) women had similar rates of disease compared to White women. Hispanic women had lower rates of endometriosis diagnosis compared to White women, although this did not reach statistical significance (RR: 0.6, 95% CI: 0.4–1.0). These findings should be interpreted cautiously as the studied population was predominantly Caucasian. Secondly, Zhao *et al.* (1998) examined the prevalence of endometriosis-related hospitalization based on the Nationwide Inpatient Sample in 1991 and 1992 in the United States. They observed significantly lower rates of endometriosis diagnosis in Black (10.1%), Hispanic (7.4%), and Asian Pacific (11.3%) women compared to White women (17.0%) (*P*  < 0.001). A more recent retrospective cohort study using claims electronic health records estimates that 70% of patients diagnosed with endometriosis were White, 6% Hispanic, 9% Asian, and 4.7% non-Hispanic Black (Christ *et al.* 2021).

This work highlighted several important themes pertinent to race/ethnicity and endometriosis. First, many of the included studies were of poor methodologic quality and at significant risk of selection bias as well as confounding, particularly from socioeconomic status. Secondly, the majority of patients (>75%) included in the studies were of White racial origin. Lastly, the majority of studies focused on disease prevalence, without exploring potential variability in presenting symptoms, diagnostic delays, or therapeutic response based on race. Literature looking at uterine fibroids, another gynecologic condition, suggests that there is significant variability amongst different ethnic groups in terms of symptom burden and clinical presentation, not necessarily in alignment with objective measures of disease burden (Murji *et al.* 2019). Flores-Caldera *et al*. recently presented the results of their international collaborative cross-sectional study which established the phenotypic profile of Hispanic/Latinx patients with endometriosis. Specifically, they identified substantial severity of symptoms, particularly dysmenorrhea and dyspareunia, high pain catastrophizing scores, and overall negative impact on quality of life (Flores-Caldera *et al.* 2021).

Finally, the primary presenting symptom of endometriosis, pelvic pain, may limit clinical consideration of this diagnosis of this condition amongst non-White patients (Alimi *et al.* 2018). Historically, medical education has perpetuated stereotypes surrounding Black patients and their experience of pain (Hoberman 2012). Significant racial and ethnic disparities remain across different areas of pain care (acute, postoperative, chronic, cancer, palliative pain), with minorities receiving lesser quality pain care than non-Hispanic white patients (Anderson *et al.* 2009). These encounters may be rooted in implicit and explicit biases held by healthcare providers including the notion that non-white patients have a higher pain threshold (Hoberman 2012, Hoffman *et al.* 2016). Similarly, when stereotypes surrounding the prevalence of diagnoses like endometriosis are perpetuated within the broader community, racialized patients may be less likely to seek medical attention for their symptoms.

## Race/ethnicity and endometriosis representation in medical education

Endometriosis and race have been discussed in medical education with heavy influence from the gynecology community. The integration of evidence-based medicine has stressed the importance of using high-quality evidence to support clinical practice; however, we must appreciate that there are many long-standing beliefs that are held as ‘mantra’ in medicine, supported mostly by flawed confirmation bias.

The perception of endometriosis as less prevalent in Black patients is widespread amongst foundational textbooks of gynecology, including but not limited to Williams Gynecology, Blueprints Obstetrics & Gynecology, and Speroff’s Clinical Gynecologic Endocrinology and Infertility (Speroff *et al.* 1983: 495–503, Fritz & Speroff 2005: 1103–1114, Callahan & Caughey 2013, Hoffman 2020). Textbooks are important to examine as they are widely distributed as educational tools based on expert opinions, and until the recent shift to online resources, formed the foundation of medical education. For example, an excerpt from the sixth edition of Novak’s Gynecology, published in 1961 states, ‘There seems no doubt that endometriosis is much more common in the white private patient than in the dispensary clientele’. (page 568) (Novak *et al.* 1961: 247–250). By the 16th edition published in 2020, the section states that endometriosis ‘is found in women from all ethnic and social groups’. (page 280) (Berek & Berek 2020). Whereas Novak’s revised its content to remove all references pertaining to race and endometriosis, other textbooks removed blatant commentary while still alluding to an ongoing ‘controversy’ (Ryan & Kistner 1999). Other examples of racial bias are more nuanced. For instance, the 2013 edition of Blueprints of Gynecology features a corresponding multiple-choice clinical vignette in which ‘Her ethnicity is Caucasian’ (page 211) is correctly identified to increase suspicion for endometriosis (Callahan & Caughey 2013). Similar commentary can be found in other gynecology textbooks dating back to the 1960s with the nature and severity of these assertions changing over time. It is of note that many of these comments are made without any appropriate citations.

## The uses and misuses of race/ethnicity in medicine

The consideration of race and ethnicity in medicine and biomedical research has been a long-standing, charged, and complex debate. There is an established history of racial injustice in medicine and a hesitance to repeat past mistakes (Phimister 2003). There are two general positions: either there is strong utility to the inclusion of race in research and medical practice or race has no biological origin and thus should not be included in medicine (Oni-Orisan *et al.* 2021). To understand each side, it is imperative to define this terminology, which has been used inconsistently. Race and ethnicity are primarily social constructs (Burchard *et al.* 2003). They arose through geographical, social, and cultural forces, as opposed to defined biologic constructs. Race is often classified based on continental origin, and historically, genetic variation has been related to geographical mating patterns (Burchard *et al.* 2003). Ethnicity is a further construct, related to geography but also considering religion, culture, and language. They are often a result of endogamous mating within continents; thus, they have genetic variation but less than continentally defined groups (Burchard *et al.* 2003). Both race and ethnicity can influence socioeconomic status, resulting in unequal access to opportunities and resources and disproportionate morbidity and mortality (Borrell *et al.* 2021).

Race/ethnicity is often used as a marker for underlying genetics. Epidemiologic and clinical research often divide participants into such categories to investigate hypotheses between environmental and genetic risk factors (Burchard *et al.* 2003). Although social determinants of health and access to care must be considered, racial/ethnic differences are often seen despite controlling for such factors. For instance, in a study by Karter *et al.* (2002), the rate of diabetic complications was evident despite using the same health maintenance organization and after adjustment for various social determinants. Conversely, while these risks are often reported as ‘intrinsic differences’ between races, they are likely capturing the risk of inequities from exposure to structural racism (Borrell *et al.* 2021).

Single gene disorders are an example of successful discovery based on race/ethnic considerations. Noting that certain groups display certain diseases, geneticists hunt for a cause, leading to discoveries of the genes for Tay-Sachs, cystic fibrosis, and thalassemia (Cooper *et al.* 2003, Phimister 2003). Mendelian disorders are often traced back to particular groups, such as Ashkenazi Jews, French Canadians, the Amish, or certain European backgrounds (Burchard *et al.* 2003), but it should be noted that these groups are not defined by race. Genetic variation is still more commonly observed within continental populations, as opposed to among them (Cooper *et al.* 2003). Common chronic diseases, such as cardiovascular disease, diabetes mellitus, and kidney disease, have been seen worldwide, confirming that all populations are susceptible, and variation is more likely due to environment. While race can help target screening for disease-associated mutations, the only way to diagnose a DNA-sequence variant is to test for it (Cooper *et al.* 2003, Phimister 2003). It should also be noted that despite all the literature on the topic, race has not been defined in genetic terms (Cooper *et al.* 2003).

Further application of race/ethnicity has been in drug responsiveness. It is well known that functional variants of genes encoding drug-metabolizing enzymes exist and understanding a patient’s background can allow clinicians to predict drug responsiveness and tailor therapies accordingly (Cooper *et al.* 2003). For instance, there has been substantial work in targeting ‘race-specific’ medical therapy in cardiovascular disease. The Clarification of Oral Anticoagulation through Genetics (COAG) trial showed a difference between the responsiveness of populations of European and African ancestry with respect to warfarin dosing, necessitating a race-specific approach to treatment (Kimmel *et al.* 2013). However, researchers must be cautious, as other randomized trials were interpreted to show different responses by race, but further analysis of the results revealed this conclusion was a type I error (Cooper *et al.* 2003). These assumptions may lead to clinicians withholding certain medications from certain racial groups, thereby exacerbating the differential care seen between groups.

While the above examples describe positive attempts at using race in medicine, there are far more examples of its misuse, of which one of the most interesting areas is in diagnostic algorithms. Used as a concrete proxy for bias, this can be seen throughout all fields of medicine. Vyas *et al.* (2020) explored some of the most common diagnostic algorithms. By including race in these calculations, they suggest that race-based medicine is being propagated, guiding decisions that may further direct resources away from minorities.

One of the most widely described algorithms incorporating race is that of estimated glomerular filtration rate (eGFR). The formula predicts higher eGFR, meaning better kidney function, for Black patients. This is supported by evidence that higher serum creatinine levels are seen in Black people, potentially due to increased muscularity (Vyas *et al.* 2020). This assumption may result in delayed referrals, and indeed Black people have higher rates of end-stage renal disease. On the other hand, ignoring race in this algorithm may lead to the prediction of worse kidney function in such patients and result in overtreatment and inappropriate drug dosing (Oni-Orisan *et al.* 2021). Similarly, the Kidney Donor Risk Index finds that black donor kidneys perform worse, and given that Black patients are more likely to receive organs from Black donors, it is not surprising that they have long wait times for renal transplantation (Vyas *et al.* 2020).

Algorithmic inclusion of race can be seen across the medical specialties. The vaginal birth after Caesarean (VBC) risk calculator predicts a lower chance of success if a patient identifies as black or Hispanic, which may deter clinicians from offering a trial of labour (Vyas *et al.* 2020). Indeed, non-White Americans have higher rates of Caesarean sections, and it is well known that Black patients have increased rates of maternal mortality (Vyas *et al.* 2020). While some algorithm developers do offer sources for these adjustments, these are often found to be outdated and biased. The racial distinctions seen in large datasets are more likely reflecting the toxic effects of racism, such as its physiological consequences, discrimination, and access to care (Vyas *et al.* 2020, Borrell *et al.* 2021). Borrell *et al.* (2021) cautions that when using standardized algorithms, clinicians should consider whether the inclusion of race would decrease health inequities, leading to better health outcomes.

The consideration of race has also led to knowledge gaps in medical research. Years of insufficient funding for research in minority populations have led to questionable generalizability of medical advances to such groups. For instance, less than 2% of National Cancer Institute-funded clinical trials and less than 4.5% of federally funded pulmonary research have included minority populations (Cooper *et al.* 2003). The National Institutes of Health requires reporting of all racial or ethnic groups, and despite this, there is a paucity of information and minimal progress in including minority groups in large trials (Burchard *et al.* 2003).

Endometriosis is another condition affected by the misuse of race. As reviewed above, it has historically been viewed as a condition of White women and a systematic review of the literature identified a strong focus of research on White women, with minimal data on minority groups (Bougie *et al.* 2019*a*, *b*). While some studies postulate that endometriosis is higher in Asian women and lower in Black women compared to their Caucasian counterparts, other studies comparing women of different races with equal indications and socioeconomic status have failed to note a difference (Kyama *et al.* 2007). It is commonly thought that those women of African descent rarely have endometriosis, yet it is one of the most common reasons for African American women in the United States to undergo gynecologic surgery (Kyama *et al.* 2007). It has also been shown that in private patients admitted for such surgeries, the prevalence of endometriosis was similar between African American and Caucasian patients (Kyama *et al.* 2007). Interestingly, Kyama *et al.* (2007) reported a significantly lower rate of endometriosis in African-Indigenous women compared to African American, indicating that race alone is not an explanation, and more likely due to lack of awareness, lack of access to laparoscopy, limited training on diagnosis and treatment, lack of research interests, and lifestyle factors (Kyama *et al.* 2007). In their retrospective study, Shade *et al.* (2012) reviewed charts of African American patients that had undergone surgery for endometriosis and noted that 93% of patients demonstrated uterine endometriotic implants. Although this was a retrospective study without a comparator group, the authors suggest that this finding may indicate a variation in disease presentation compared to other racial/ethnic groups.

The debate regarding the use and misuse of race/ethnicity in medicine is ongoing and fraught with complexities. While race is not a reliable proxy for genetic difference, we must acknowledge that differences do exist between people of different racial categories and this is clinically meaningful (Oni-Orisan *et al.* 2021). Ultimately, replacing race with genetic ancestry would result in more informative, evidence-based approaches, yet this technology is not yet readily available outside the research environment (Wadman 2004, Oni-Orisan *et al.* 2021). While ignoring race may improve equality, it is only through the equity that racial disparities can be tackled (Oni-Orisan *et al.* 2021).

## Phenomics and endometriosis

The advent of genome-wide association studies (GWAS) has gathered a great amount of information suggesting high-confidence genotype–phenotype associations between specific genomic loci and a large number of diseases, including diabetes, obesity, Crohn’s disease, and hypertension. Recognizing the importance of considering phenotypic variation among endometriosis patients, Vigano *et al.* (2012) conducted a comprehensive review of the relationship between morphometric traits and endometriosis. They identified some association between BMI and particularly pigmentary trains/presence of nevi and diagnosis of endometriosis. More importantly, they drew attention to the consideration of genomic contribution to the phenotype of endometriosis. Although currently the mechanisms underlying genotype–phenotype relationships remain only partially explained and must be interpreted in the context of multiple limitations, including the inherent variability in quality of published data and the higher order complexity of the genotype–phenotype relationship, it is easily imaginable that a catalogue of nearly all human genomic variations and their relative impact on human diseases will be available within our lifetime (Vidal *et al.* 2011). We must appreciate that even with expansion of genomic information that will become available, the majority of phenotypic variation seen amongst endometriosis and other medical conditions will stem from genotype–environment interaction (Vidal *et al.* 2011). Genetic variation (although there is not much) between populations tends to be geographically structured, as expected from the partial isolation of human populations during much of their history (Jorde & Wooding 2004). In this regard, race may be considered first-order approximation to the geographically structured phenotypic variation in the human species (Relethford 2009). The influence of environmental exposure, resulting from geographic variation, on the development of endometriosis has been suggested (Soave *et al.* 2015).

## Conclusions and future work

In summary, we see that there is a strong historical bias regarding the epidemiological impact of race/ethnicity on the prevalence of endometriosis. Although the summation of current evidence suggests that endometriosis may be less common amongst Black and Hispanic women, compared to White women, it is imperative to recognize the significant methodological flaws and bias driving the studies performed to date. Furthermore, it is important to question the relevance of this information in the provision of outstanding and individualized patient care. Recognizing the narrative on this topic to date, we suggest the following clinical, education, and research priorities moving forward:

1. Healthcare providers of patients presenting with symptoms associated with endometriosis, including but not limited to dysmenorrhea, pelvic pain, and infertility, consider this diagnosis in all patients, regardless of their race/ethnicity. Providers should reflect on their own potential implicit and explicit bias regarding this topic and if necessary, consult resources to help mitigate biased beliefs. Patients and providers should consider that phenotypical characteristics of patients with endometriosis may involve different symptoms, disease presentation, and comorbidities (Vigano *et al.* 2012).
2. In recognition of the racial bias still pervasive in medical education materials, intervention is necessary to limit misinformation learners may carry into their clinical work and future practice. This will require institutions and organizational bodies to invest appropriate resources into critical review of current assertions on endometriosis. Revisions to medical education, including but not limited to textbooks, curricula, and licensing exams, should include a diverse representation of educators, particularly under-represented clinicians, which may include racialized persons, people living with disabilities, and those who identify as LGBTQ2+. Additionally, relevant stakeholders should develop critical guidelines on the discussion of race in medical education. Racial disparities should be discussed with citations from credible research as well as with potential systemic origins for such disparities (Amutah *et al.* 2021). Moreover, it is important to note that race should not be used as a proxy for biological markers or genetic predisposition for a disease (Vyas *et al.* 2020, Amutah *et al.* 2021). Race also should not be used to simplify systemic contributors when teaching around the underlying cause for disparities in diagnoses (Williams *et al.* 2010). Medical trainees must be taught social determinants of health in order to holistically serve diverse patient populations.
3. Further investigation into the epidemiologic risk factors predisposing the development of endometriosis should be encouraged in order to identify at-risk individuals and implement early detection and appropriate treatment of the condition. Research exploring the unique presentation and treatment of endometriosis amongst patients may explore race and ethnicity but needs to include these factors in a sensitive manner, reflecting on underlying social constructs. Any researcher including race/ethnicity when studying endometriosis should reflect *a priori* on the reason and implications of including this factor in their study.

We advocate for the adaptation of an individualized and patient-centred approach to the management of endometriosis to achieve more equitable and improved care provision for all endometriosis patients.

## Declaration of interest

O Bougie has participated in a speakers’ bureau and received research grants and consulting fees from Bayer Pharma, Allergan, Hologic and AbbVie. The authors confirm these sponsorships had no involvement in the study. I N and C W have no disclosures.

## Funding

This work did not receive any specific grant from any funding agency in the public, commercial, or not-for-profit sector.

## Author contribution statement

All authors (O B, I N and C W) contributed to the research and manuscript preparation. They all approved the final submitted version.
